# Additively Manufactured Flexible Electronics with Ultrabroad Range and High Sensitivity for Multiple Physiological Signals' Detection

**DOI:** 10.34133/2022/9871489

**Published:** 2022-08-05

**Authors:** Huanhuan Feng, Yaming Liu, Liang Feng, Limeng Zhan, Shuaishuai Meng, Hongjun Ji, Jiaheng Zhang, Mingyu Li, Peng He, Weiwei Zhao, Jun Wei

**Affiliations:** ^1^Sauvage Laboratory for Smart Materials, Shenzhen Key Laboratory of Flexible Printed Electronics Technology, Harbin Institute of Technology (Shenzhen), China; ^2^State Key Laboratory of Advanced Welding and Joining (Shenzhen), Harbin Institute of Technology (Shenzhen), China; ^3^State Key Laboratory of Advanced Welding and Joining, Harbin Institute of Technology, China

## Abstract

Flexible electronics can be seamlessly attached to human skin and used for various purposes, such as pulse monitoring, pressure measurement, tensile sensing, and motion detection. Despite their broad applications, most flexible electronics do not possess both high sensitivity and wide detection range simultaneously; their sensitivity drops rapidly when they are subjected to even just medium pressure. In this study, ultrabroad-range, high-sensitivity flexible electronics are fabricated through additive manufacturing to address this issue. The key to possess high sensitivity and a wide detection range simultaneously is to fabricate flexible electronics with large depth-width ratio circuit channels using the additive manufacturing inner-rinsing template method. These electronics exhibit an unprecedented high sensitivity of 320 kPa^−1^ over the whole detection range, which ranges from 0.3 to 30,000 Pa (five orders of magnitude). Their minimum detectable weight is 0.02 g (the weight of a fly), which is comparable with human skin. They can stretch to over 500% strain without breaking and show no tensile fatigue after 1000 repetitions of stretching to 100% strain. A highly sensitive and flexible electronic epidermal pulse monitor is fabricated to detect multiple physiological signals, such as pulse signal, breathing rhythm, and real-time beat-to-beat cuffless blood pressure. All of these signals can be obtained simultaneously for detailed health detection and monitoring. The fabrication method does not involve complex expensive equipment or complicated operational processes, so it is especially suitable for the fabrication of large-area, complex flexible electronics. We believe this approach will pave the way for the application of flexible electronics in biomedical detection and health monitoring.

## 1. Introduction

Flexible electronics are undergoing major advancements and are being widely used in many applications [1–3], such as pulse monitoring, pressure measurement, tensile sensing, and motion detection. Unfortunately, most flexible electronics do not possess both high sensitivity and wide detection range simultaneously [1–3]. Their detection range is typically very narrow, and their sensitivity drops rapidly when they are under considerable or even medium pressure [4–13], which limits their application in flexible sensors [14, 15], integrated e-skin [16, 17], human health monitoring equipment [18–20], and nerve interfaces [21].

Many attempts have been made to address this issue; most of them are using liquid metal alloy eutectic gallium indium (EGaIn) as the circuit building material [14–22]. Flexible electronics can be categorized into three types as their dimensions increase from one-dimensional (1D) to three-dimensional (3D) [14, 23–26]. Regarding 1D flexible electronics, Liu's group coated polyethylene terephthalate/polybutylene terephthalate (PET/PBT) fiber with liquid metal to obtain super stretched conductive fiber [27]. Such 1D conductive fibers can be produced quickly at low cost, but their pressure detection range is only from 0.001 to 0.009 (×10^−2^ N), and their sensitivity is around 0.5 kPa^−1^. Regarding 2D flexible electronics, there are several options to obtain 2D liquid metal flexible electronics, including the laser-engraving method [23], photolithographic method [28], coaxial writing method [29], printing method [24] (including noncontact printing [30–32] and contact printing [25, 33, 34]), and Cu trace wetting method [35]. The performance of 2D flexible electronics can be fine-tuned as needed, but they cannot be attached to human skin seamlessly. Their pressure-sensing range is also narrow (1.5 N–3.5 N), and their sensitivities are less than 1 kPa^−1^.

Regarding 3D flexible electronics, various flexible electronics [29, 36–38] have been obtained by filling liquid metal into various 3D structures made from elastomers. Yao's group induced liquid metal into polydimethylsiloxane (PDMS) foam (cubic sugar template) to create a liquid metal network [36]. This procedure can be used to obtain a porous substrate filled with liquid metal, and the resistance of the product will change in response to deformation. However, this procedure cannot be used to build a custom circuit as needed. Zhang's group showed that liquid metal circuits with custom 3D structures could be constructed using a method in which liquid metal penetrates and diffuses into tunable granular porous materials spontaneously by capillary force [37]. Using this method, they were able to build 3D liquid metal filled foam structures as needed, and they were able to fine-tune the performance of the structures. The only weakness to this method was the poor stretchability of the structures. Seo's group successfully fabricated flexible liquid metal circuits with high-precision patterns by one-step ink-jet deposition using lithographic mold masks [39]. Their method can be used to manufacture complex 3D flexible circuits with very high resolution (30 *μ*m), which can detect a force (12 N) oscillation with a maximum frequency of 4 Hz. Unfortunately, Seo's one step coating method is too complex, and the equipment is too expensive for small research groups to afford. Although these previous studies have achieved great progress in liquid metal flexible electronics fabrication and application, problems remain including expensive equipment, complex procedures, and poor pressure-sensing performance. MXene/tissue paper sensors and self-powered sensors are also high-performance flexible sensors, and they have drawn considerable attention [40, 41]. They have low production costs, low energy consumption, and minimal environmental impact [4, 42, 43].

To address these problems, we demonstrate a facile additive manufacturing approach for ultrabroad-range, high-sensitivity flexible electronics. Via this approach, hollow 3D tubes are additively manufactured with an ABS (acrylonitrile butadiene styrene) 3D printer. Surface modification is finalized by sputtering an Au layer with 30 nm thickness. The tubes are then easily transferred into the flexible circuit by dissolving ABS with acetone inner rinsing and liquid metal filling. No clogging occurs during the acetone inner rinsing through the hollow ABS tubes. The sputtered metal layers on the 3D sacrificial template surface turn into the inner-layers of the microchannel, which facilitate liquid metal filling later. The fabrication does not involve expensive equipment or a complicated operation process, and it is especially suitable at fabricating large-area, complex flexible circuits. Our flexible electronics exhibit outstanding performance compared with previous reports [14, 23–26]. Their high sensitivity of 320 kPa^−1^ over the whole detection range, which is from 0.3 to 30,000 Pa (five orders of magnitude), is unprecedented. Their detection limit can reach as little as 0.02 g (the weight of a fly), which is comparable with human skin. They also show excellent stretchability, as they can endure a strain over 500% without breaking. Furthermore, they possess very good mechanical durability and stability with negligible drift (4.8%) after 1000 repetitions of stretching to 100% strain.

A highly sensitive flexible electronic epidermal pulse monitor is fabricated to detect multiple subtle physiological signals, such as breathing rhythm, pulse signal, and real-time beat-to-beat cuffless blood pressure (BP). All of the signals can be obtained simultaneously. We believe this approach will pave the way for the wider application of flexible electronics in biomedical detection and health monitoring.

## 2. Flexible Electronics Fabrication

**Figure 1:**
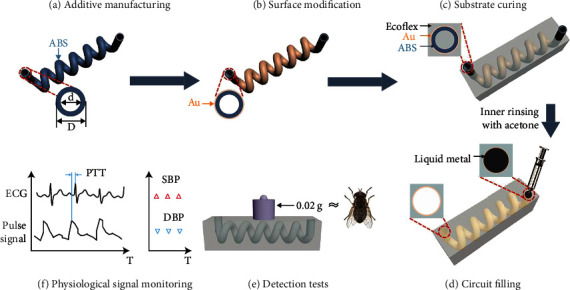
Schema of the flexible electronics' fabrication and application. (a) Additive manufacturing hollow tube as 3D sacrificial template. (b) Surface modification via sputtering metal, namely Au. (c) Curing Ecoflex substrate and inner rinsing with acetone. (d) Circuit filling with liquid metal. (e) Detection testing in terms of range and limitation. (f) Pulse and blood pressure detection and real-time monitoring.

A hollow tube is fabricated with a 4 mm outer diameter, 2 mm inner diameter, and 1 mm thickness (Figure 1(a)). Then, an Au layer is sputtered on its surface with an optimized thickness of 30 nm (Figure 1(b)). Next, the Au sputtered hollow tube is dipped into Ecoflex, and the Ecoflex substrate is cured at room temperature within 1 hour (Figure 1(c)). The hollow tube is then dissolved with acetone by inner rinsing through the hollow tube to obtain a built-in microchannel. The sputtered Au layer becomes the inner-layer of the microchannel, which facilitates liquid metal filling. The flexible electronics are obtained after EGaIn is filled into the microchannels (Figure 1(d)). During detection, the liquid metal will be deformed, and its resistance will increase with increasing pressure. The weight detection experimental results show that the minimum detectable weight is 0.02 g, which is equivalent to the weight of a fly (Figure 1(e)). This means our flexible electronics possess sensitivity similar to the human skin [44]. A highly sensitive flexible electronics epidermal pulse monitor is fabricated to detect multiple subtle physiological signals, such as pulse signal, breathing rhythm, and real-time beat-to-beat cuffless BP. The beat-to-beat systolic blood pressure (SBP) and diastolic blood pressure (DBP) are obtained by synchronizing epidermal pulse and electrocardiogram (ECG) signals using the pulse transit time (PTT) method (Figure 1(f)). BP values are consistent with commercial BP detector measurement.

## 3. Flexible Electronics Mechanical Characterization

**Figure 2:**
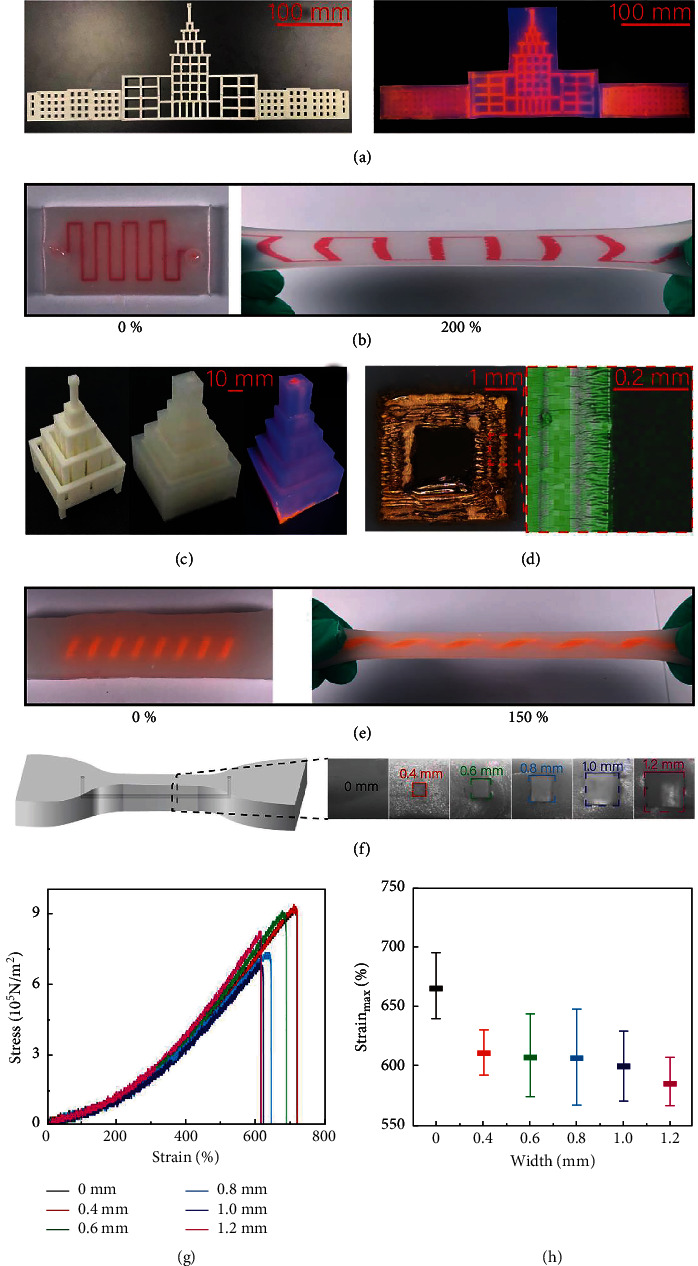
Structure and mechanical property characterization of flexible electronics. (a) 2D model of main building of Harbin Institute of Technology (HIT). (b) Stretching of 2D channel. (c) 3D model of HIT main building. (d) Scanning electron microscope (SEM) image of hollow tube cross-section. (e) Stretching of 3D spiral channel. (f) Model of tensile tests with different circuit diameters. (g) Individual tensile tests of flexible electronics with different circuit diameters. (h) Statistically averaged tensile tests of flexible electronics with different circuit diameters.

The structural manufacturing and mechanical performance are illustrated in Figure 2. All the scale bar units are in millimeters. The 2D sacrificial template of the main building of the Harbin Institute of Technology (HIT) is shown in Figure 2(a) (length 500 mm, height 350 mm, and thickness 4 mm). The left side of Figure 2(a) shows the sacrificial template, and the right side of Figure 2(a) shows the template covered with Ecoflex and filled with fluorescent dye (methyl red) with UV excitation. This is one of the largest existing 2D structures created with 2D printing technologies. Figure 2(b) shows a 2D circuit and its stretchability with 200% strain. Figure 2(c) shows the largest existing 3D model of the main building of HIT (length 600 mm, height 150 mm, and thickness 60 mm). This is one of the largest existing 3D structures created via 3D printing technologies [29]. The cross-sectional view of one of the hollow tubes is shown in Figure 2(d). The outside diameter is 4 mm, the inner diameter is 2 mm, and the tube thickness is 1 mm. The left side of Figure 2(d) is a scanning electron microscope (SEM) image, and the right side of Figure 2(d) is zoomed-in energy-dispersive X-ray spectroscopy elemental mapping. The microchannel is quite smooth compared to other methods [45]. As shown in Figure 2(e), a 3D helix structure is also fabricated and stretched to 150% strain. The mechanical tests depicted in Figure 2(f) are performed on Ecoflex samples of a specific shape with dimensions of 5 mm × 5 mm × 20 mm and an inner diameter varying from 0 to 1.2 mm. The breaking strain decreases from 720% to 610% as the inner diameter varies from 0 to 1.2 mm, as shown in Figure 2(g). As shown in Figure 2(h), the average breaking strain decreases from 670% to 570% as the inner diameter varies from 0 to 1.2 mm. The difference in breaking strain increases (22% to 20% to 34%) as the inner diameter increases from 0 mm to 0.8 mm. Then the difference in breaking strain decreases (34% to 30% to 25%) when the inner diameter exceeds 0.8 mm. All the 3D flexible electronics samples can be stretched 570% without breaking. The performance of the flexible electronics is much better than specified by the application requirements since the maximum stretching requirement applied to human tissues or organs is usually 100%. The resultant Ecoflex with a built-in 3D structure channel has superior stretchability and flexibility compared to existing flexible electronics.

## 4. Flexible Electronics Electrical Characterization

**Figure 3:**
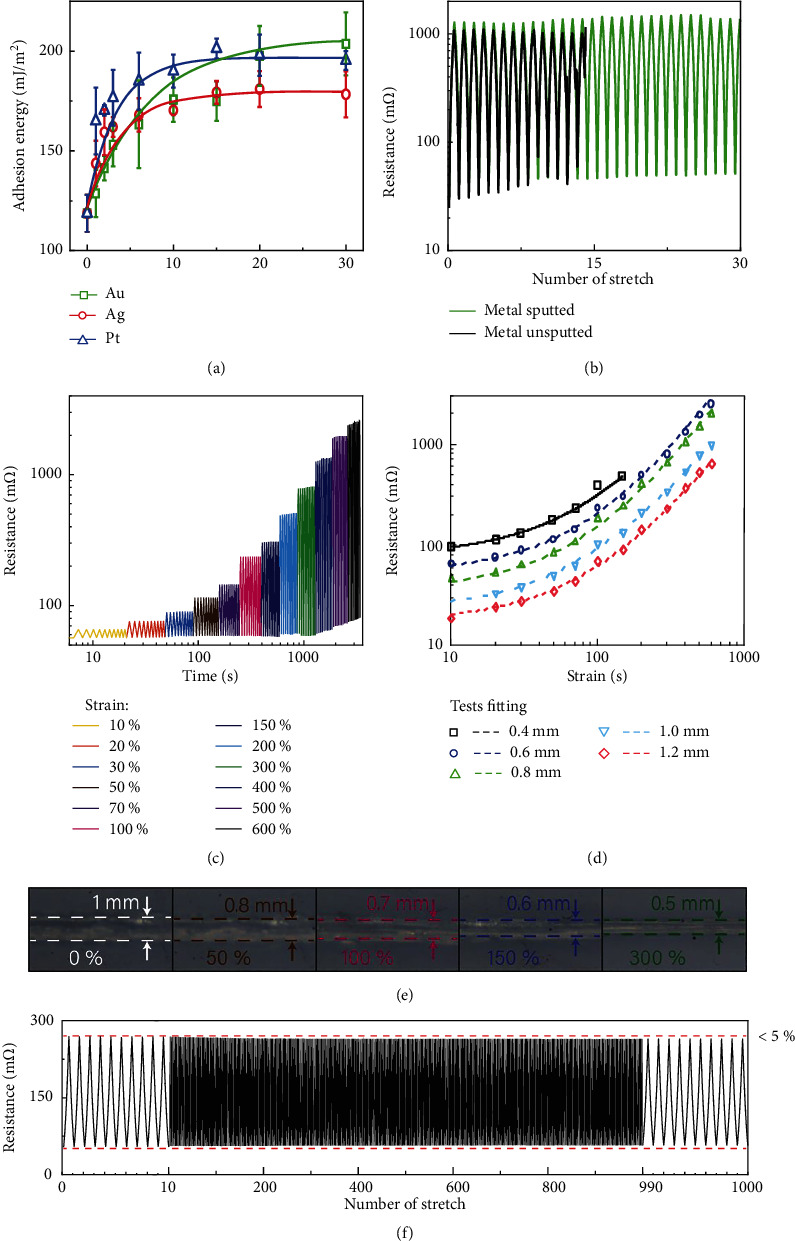
Electrical characterizations of the 3D flexible electronics. (a) The experimental surface tension and the theoretical surface tension after metal sputtering. (b) Stabilities of devices with channel metal sputtered and unsputtered. (c) Repeated stretch tests under different strains, namely 10%, 20%, 30%, 50%, 70%, 100%, 200%, 300%, 400%, 500%, and 600%. (d) The experimental and theoretical resistance values of sensor's resistance under tensile strain. (e) The stretching circuit channel. (f) Tactile durability test of Ecoflex sensor under a strain of 100% at a frequency of 0.1 Hz. The resistance change curves were recorded after every 1000 cycles, and 10 cycles of data are presented in each record.

The surface of Ecoflex is sputtered with Au, Ag, and Pt separately to improve the surface wettability. As the thickness of the sputtering layer increases, the surface static contact angle decreases, whereas the surface rolling angle and surface covering area increase. The details are shown in Supplementary Figures 1–4. The results indicate that the metal coverage reaches saturation at a sputtering thickness of 30 nm for Au, Ag, and Pt. Au has the best wettability among them. The surface adhesion energy of Au as a function of time is shown in Figure 3(a). The flexible sensors with/without Au inner-layer are stretched to 600% strain repeatedly to test the improvement of stretch durability. The results show the flexible sensors with an Au inner-layer can endure 30 repetitions, which is about twice the number of repetitions endured without an Au inner-layer (Figure 3(b)). As shown in Figure 3(c), the samples are stretched 120 times with increasing strain from 10 to 600%. The resistance changes as the strain varies. The average peak value fits very well with the calculation result of formula (1), as shown in Figure 3(d). The derivation of the formula can be found in Supplementary Fig. 5. Our samples exhibit a very good recovery performance, as shown in Supplementary Fig. 6. There is little hysteresis in consecutive loading-unloading cycles. The determination of resistance is derived by the following formula:

(1) $$\begin{array}{c} R=R_{0}{\left(1+\frac{L}{L_{0}}\right)}^{2}, \end{array}$$

where *R* is the current resistance, *R*_0_ is the initial resistance, *L* is the current length, and *L*_0_ is the initial length.

In addition, we also measure the channels' width under different strains (Figure 3(e)), and the results follow a derivation formula:

(2) $$\begin{array}{c} a=\sqrt{\frac{a_{0}^{2}}{\left(1+\varepsilon \right)}}, \end{array}$$

where *a* is the current width of the channel, *a*_0_ is the initial width of the channel, and *ε* is the strain of the electronic. Because the response of the equipment to strain strictly follows the resistance deformation formula, the device can be used to detect bending and twisting deformation (Supplementary Fig. 7 and Fig. 8).

Our flexible electronics can endure 1000 repetitions of stretching to 100% strain and show no mechanical fatigue. The relative resistance changes are less than 5% (Figure 3(f)). The electronic device has great durability, which could be attributed to the very smooth microchannel and the better liquid metal wettability to sputtered Au. The results show that the flexible sensor has a very sensitive response and good stability compared to other flexible sensors [46, 47]. Thus, the flexible electronic system fully meets the requirements for monitoring complex physiological signals of humans.

## 5. Multiple Physiological Signals Detection

**Figure 4:**
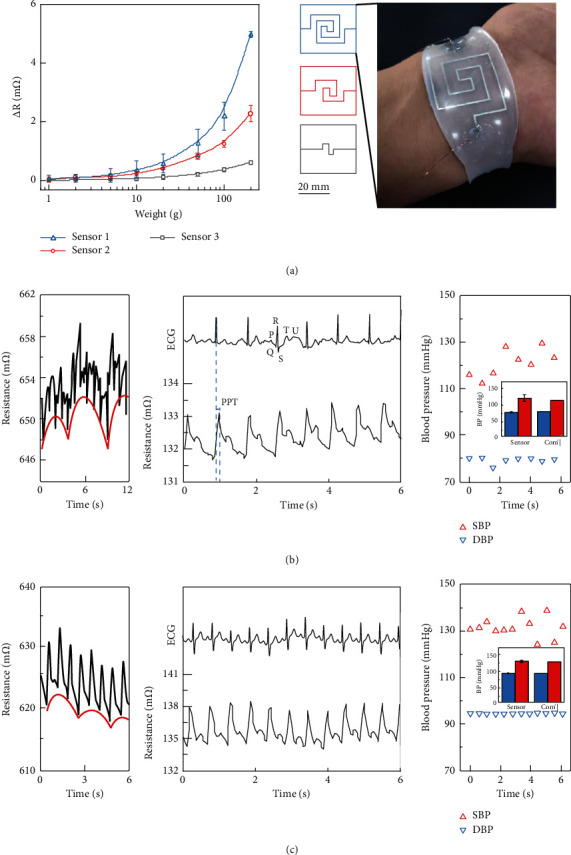
Sensor for real-time blood pressure monitoring. (a) The response of different sensors to pressure and designed channel shape and the real sensor attached to the volunteers' wrists. (b) Pulse signal measured by sensor 1 at the quiescence state and beat-to-beat systolic blood pressure (SBP) and diastolic blood pressure (DBP) calculated from the ECG and epidermal pulse signals. (c) Pulse signal measured by sensor 1 after intensive activity and beat-to-beat SBP and DBP calculated from the ECG and epidermal pulse signals.

The sensing accuracy is improved by increasing the length of the liquid metal circuit (from 7.2 mm/cm^2^, to 13.6 mm/cm^2^, to 23.2 mm/cm^2^), as shown as Figure 4(a). The devices can be used to detect and monitor multiple physiological signals, such as breathing, pulse, and BP [26]. As shown in Figure 4(b), 1, the respiratory rate of the human body at the quiescence state can be measured by the flexible sensor and tends to be around 13 breaths per minute. As shown in Figure 4(b), 2, the signal of the sensor at the quiescence state has a lower heart rate (72 ppm) and lower amplitude (133 m*Ω*). As shown in Figure 4(c), 1, the respiratory rate of the human body after intensive activity can be measured by the flexible sensor and tends to be around 30 breaths per minute. As shown in Figure 4(c), 2, the signal of the sensor has a faster heart rate (112 ppm) and higher amplitude (138 m*Ω*). The characteristic peaks of specific pulse waves can also be obtained, including main peak and *f* waves. After the pulse signal is synchronized with the epidermal electrocardiogram (ECG) signal, the pulse transit time (PTT)—which is the time difference between the main pulse peak and the main ECG peak—can be obtained. The beat-to-beat systolic blood pressure (SBP) and diastolic blood pressure (DBP) are obtained by synchronizing epidermal pulse and epidermal ECG signals using the PTT method. The SBP and DBP at each pulse beat can be calculated according to the PTT by the following formulas, respectively:

(3) $$\begin{array}{c} DBP=\frac{{SBP}_{0}}{3}+\frac{2{DBP}_{0}}{3}+\text{Aln}\left(\frac{{PTT}_{0}}{PTT}\right)-\frac{\left({SBP}_{0}-{DBP}_{0}\right)}{3}\frac{{PTT}_{0}^{2}}{{PTT}^{2}}, \end{array}$$

(4) $$\begin{array}{c} SBP=DBP+\left({SBP}_{0}-{DBP}_{0}\right)\frac{{PTT}_{0}^{2}}{{PTT}^{2}}, \end{array}$$

where *PTT*_0_ is the *PTT* of the first recorded cardiac cycle and *A* is a subject-dependent coefficient but can be approximated as a constant [48]. The PTT method requires a one-time calibration procedure where a standard clinical device (e.g., a sphygmomanometer) is applied together with a cuffless BP sensor system to determine the initial *SBP*_0_ and *DBP*_0_ of a volunteer. The DBP of a volunteer at the quiescence state is 78.96 ± 1.34 mmHg, and the SBP is 114.93 ± 2.98 mmHg. The DBP of a volunteer after intensive activity is 94.47 ± 0.21 mmHg, and the SBP is 132.39 ± 4.46 mmHg. The DBP and SBP of a volunteer at the quiescence state measured by the commercial sphygmomanometer were 79 mmHg and 116 mmHg, respectively. The DBP and SBP of a volunteer after intensive activity measured by the commercial sphygmomanometer were 94 mmHg and 139 mmHg, respectively. The deviation between the blood pressure measured using our sensor combined with the PPT method and the blood pressure measured using the commercial sphygmomanometer was never more than 5%.

This approach is noninvasive and convenient compared with the traditional method, which evolves large instruments and a complex procedure. Multiple physiological signals, such as pulse signal, breathing rhythm, and real-time beat-to-beat cuffless BP, can be obtained simultaneously, which is vital in clinical scenarios such as spotting symptoms of SARS-CoV-2 infection [48] or detecting pain [49].

## 6. High Sensitivity in Wide Detection Range

**Figure 5:**
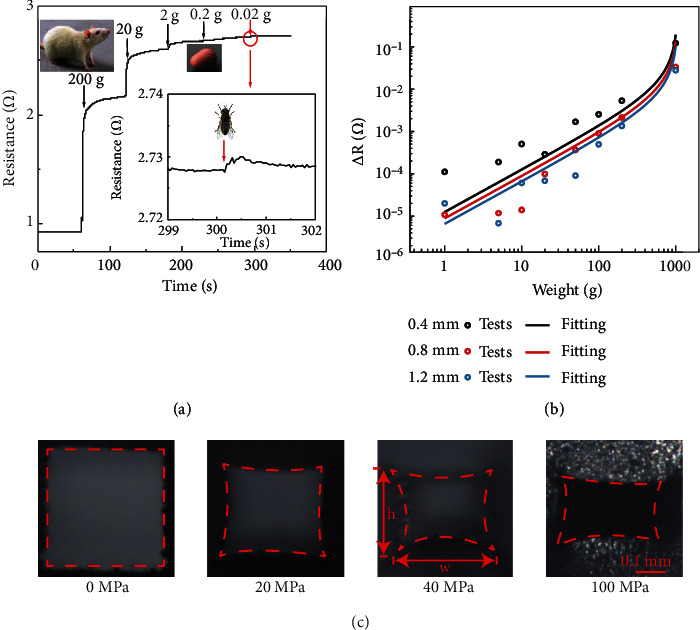
Ultrahigh sensitivity and theoretical modelling. (a) Minimum pressure threshold of sensor 1. (b) The response of the sensor to pressure under different quantities of tension. (c) Changes in channel cross-section during pressure.

As shown in Figure 5(a), different weights (200 g, 20 g, 2 g, 0.2 g, and 0.02 g) are superimposed on the sensor every 60 s, and the resistance change is continuously measured. In the process of applying pressure, the cross-sectional area decreases, so the sensitivity increases. The detection range reaches five orders of magnitude, and the resolution of pressure is 0.3136 Pa, which corresponds to the weight of a fly. The reliable signal change can be read when it is three times greater than the noise. As shown in Figure 5(b), the results also conform to the electrical resistance change formula mentioned in Wood's study [50]:

(5) $$\begin{array}{c} ∆R=\frac{\rho L}{wh}\left\{\frac{1}{1-2\left(1-v^{2}w\chi p/Eh\right)}\right\}, \end{array}$$

where *v* is the Poisson's ratio of Ecoflex, *E* is the elastic modulus of Ecoflex, *p* is the pressure applied to the sensor, *L*, *w*, and *h* are the length, width, and height of the sensor circuit channel, respectively, and *χ* is the correction constant. The results show that our sensor has the same sensitivity as the human skin. In addition, in the process of stretching, the cross-section of the EGaIn flexible circuit per unit area becomes narrower, and the deformation produced by the same pressure increases; thus, the sensitivity increases (Figure 5(c)).

Subsequently, five kinds of sensors with different channel sizes and three kinds of patterns are utilized to conduct gradient weighting experiments. The results show that the sensitivity decreases as the channel size increases (Supplementary Fig. 9). Additionally, five kinds of sensors with different Ecoflex substrate thicknesses and three kinds of patterns are utilized to conduct gradient weighting experiments. The results show that the sensitivity decreases as the Ecoflex substrate thickness increases (Supplementary Fig. 10). Therefore, it can be predicted that the higher the degree of EGaIn folding, the longer the length of EGaIn wire per unit area or volume, and the greater the resistance increment under the same degree of deformation. The introduction of a 3D structure can maximize the length of EGaIn wire per unit area.

**Figure 6:**
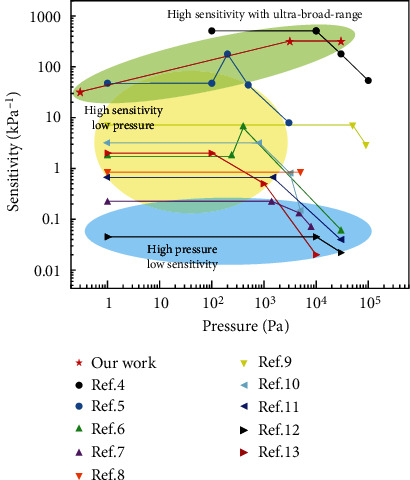
Comparison of sensitivity and detection range of related sensors [4–13].

Many researchers have explored the manufacturing of sensors for health monitoring, as summarized in Figure 6. As shown in the Figure 6, our flexible sensor has a pressure sensitivity of 32 kPa^−1^ in the range of 0.3-3000 Pa and 320 kPa^−1^ in the range of 3000-30000 Pa; it shows very good sensitivity and wide flexible sensing range. And different from the traditional flexible sensor, our flexible sensor will become more sensitive when the pressure increases. Therefore, our flexible sensor can be used to detect micro pressure under high pressure.

## 7. Conclusions

We have reported flexible electronics with high sensitivity and ultrabroad pressure detection range that can be made by 3D printing a hollow sacrificial template and combining the template with EGaIn. The flexible sensor generated via the above method has superior performance compared to existing flexible sensors. Its sensitivity to pressure detection is close to that of human skin, so it is expected to be applied to electronic skin. Via the flexible sensor, a pulse can be detected with fine resolution, and BP can be detected in real time combined with ECG. In comparison with other EGaIn-based flexible sensors, the fabrication method of our flexible sensor does not involve complex expensive equipment or complicated operational processes, so it is especially suitable for the fabrication of large-area, complex flexible electronics. Our sensors have excellent performance, offering high sensitivity and an ultrabroad pressure detection range compared with existing approaches. Our sensors therefore have outstanding potential for health monitoring, as they can simultaneously detect multiple physiological signals. We believe our fabrication method can play an important part in the future fabrication of multifunctional e-skin with high sensitivity and wide detection range.

## 8. Methods

### 8.1. Ultrasensitive Flexible Electronic Sensor Fabrication

ABS is squeezed out by a nozzle at 250°C, the temperature of the receiving plate is 70°C, the squeezing speed is 40 mm/s, and the distance between the nozzle and plate is 0.2 mm. Alternatively, other 3D printing processes with submicrometer resolution can be considered for this step. The sacrificial template is sputtered with Au for 300 seconds by an ion sputtering instrument (Beijing Zhongkekeyi SBC-12). The sacrificial template is immersed into liquid Ecoflex, and liquid Ecoflex is cured into solid Ecoflex at room temperature for 1 h. The solid Ecoflex is immersed in an acetone solution so that the ABS template inside it dissolves. Then the Ecoflex template is rinsed well with deionized water and dried by a drying machine (Shanghai Yiheng S1900378). EGaIn (Shuochen Metal) is purchased from the market. EGaIn is perfused into the channel, 90 mm Cu wires are connected to both ends of the EGaIn pattern, and the EGaIn pattern is sealed with liquid Ecoflex.

### 8.2. Mechanical Properties Test

All mechanical tests are performed using a force tester (Mark-10 ESM303). All samples are cut into dumbbell shapes, which are 5 mm in thickness, 5 mm in width, 20 mm in length, and 5 mm in gauge length and width. Samples with six different channel sizes (i.e., 0 mm, 0.4 mm, 0.6 mm. 0.8 mm, 1.0 mm, and 1.2 mm in diameter), all of which are 10 mm in length, are created.

### 8.3. Electrical Properties Test

To test electrical properties, a custom-built mechanical translation stage (Zolix) coupled with a multimeter (Keithley DMM7510) is used to measure device resistance.

### 8.4. Sensor for Blood Pressure Measurement

To detect BP, the sensor is set on the volunteer's right wrist, and at the same time, a smart wristwatch (HUAWEI VID-B99) is set on the volunteer's left wrist to detect the ECG signal. A multimeter (Keithley DMM7510) is used to test the resistance of the sensor. A commercial sphygmomanometer is used to detect the volunteer's BP.

### 8.5. Sensor for E-Skin

To detect the sensor's pressure detection limit, a multimeter (Keithley DMM7510) is used to test the resistance of the sensor, and 12 weights (i.e., 200 g, 100 g, 50 g, 20 g, 10 g, 5 g, 2 g, 1 g, 0.2 g, 0.1 g, 0.05 g, and 0.02 g) are used to apply pressure.

## Data Availability

The authors declare that main data supporting the findings of this study are available within the paper and its supplementary information files. Extra data are available from the corresponding authors upon reasonable request.

## Supplementary Materials

Supplementary 1 Figure S1: Au, Ag, and Pt adhere on Ecoflex surface. Figure S2: Au, Ag, and Pt layer covering area versus sputtering layer thickness. Figure S3: static contact angle of EGaIn on Ecoflex surface. Metal sputtering is carried out with Au, Ag, and Pt separately. Metal sputtering times are 0, 10, 20, 30, 60, 100, 150, 200, and 300 seconds. Figure S4: sliding angle of EGaIn on Ecoflex surface. Metal sputtering is carried out with Au, Ag, and Pt separately. Metal sputtering times are 0, 10, 20, 30, 60, 100, 150, 200, and 300 seconds. Figure S5: theoretical model of resistance variation as a function of elongation *ε*. Figure S6: loading–unloading experiments under different degrees of tension. Figure S7: bending tests and schematic diagram. Figure S8: twisting test and schematic diagram. Figure S9: effect of channel size on circuit sensitivity. Figure S10: effect of substrate thickness on circuit sensitivity.

Supplementary 2 Movie S1: multiple physiological signals detection and monitoring.
