# Supplementary material for: Additively Manufactured Flexible Electronics with Ultrabroad Range and High Sensitivity for Multiple Physiological Signals' Detection
Source: Research (Wash D C). 2022 Aug 5;2022:9871489. doi: 10.34133/2022/9871489 (PMC9394051; doi:10.34133/2022/9871489)
Supplement: Supplementary 1 — Figure S1: Au, Ag, and Pt adhere on Ecoflex surface. Figure S2: Au, Ag, and Pt layer covering area versus sputtering layer thickness. Figure S3: static contact angle of EGaIn on Ecoflex surface. Metal sputtering is carried out with Au, Ag, and Pt separately. Metal sputtering times are 0, 10, 20, 30, 60, 100, 150, 200, and 300 seconds. Figure S4: sliding angle of EGaIn on Ecoflex surface. Metal sputtering is carried out with Au, Ag, and Pt separately. Metal sputtering times are 0, 10, 20, 30, 60, 100, 150, 200, and 300 seconds. Figure S5: theoretical model of resistance variation as a function of elongation ε. Figure S6: loading–unloading experiments under different degrees of tension. Figure S7: bending tests and schematic diagram. Figure S8: twisting test and schematic diagram. Figure S9: effect of channel size on circuit sensitivity. Figure S10: effect of substrate thickness on circuit sensitivity. [file 9871489.f1.docx]

# Additively manufactured flexible electronics with ultra-broad range and high sensitivity for detection of multiple physiological signals

Huanhuan Feng¦*^a,b^, Yaming Liu¦^a,b,c^, Liang Feng^a,b^, Limeng Zhan^a,b^, Shuaishuai Meng^a,b^,

Hongjun Ji^a,b^, Jiaheng Zhang^a,b^, Mingyu Li^a,b^, Peng He*^c^, Weiwei Zhao*^a,b^, Jun Wei*^a,b^

a. Sauvage Laboratory for Smart Materials, Shenzhen Key Laboratory of Flexible Printed Electronics Technology, Harbin Institute of Technology (Shenzhen), China

b. State Key Laboratory of Advanced Welding and Joining (Shenzhen), Harbin Institute of Technology (Shenzhen), China

c. State Key Laboratory of Advanced Welding and Joining, Harbin Institute of Technology, China

## Microscopy, SEM, and EDS of Au, Ag, and Pt sputtered on Ecoflex substrate


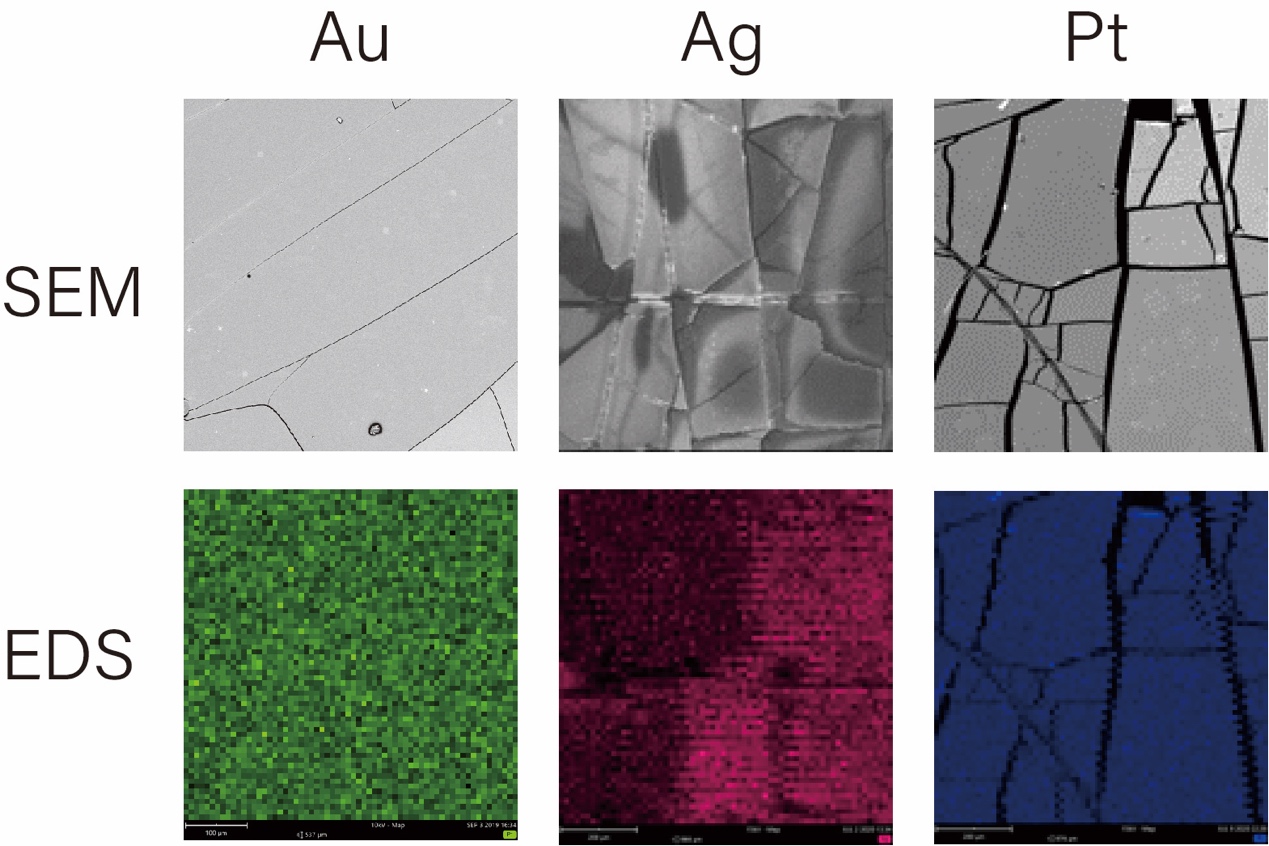


Figure S1. Au, Ag, and Pt adhere on Ecoflex surface.

## Metal layer covering area versus sputtering layer thickness


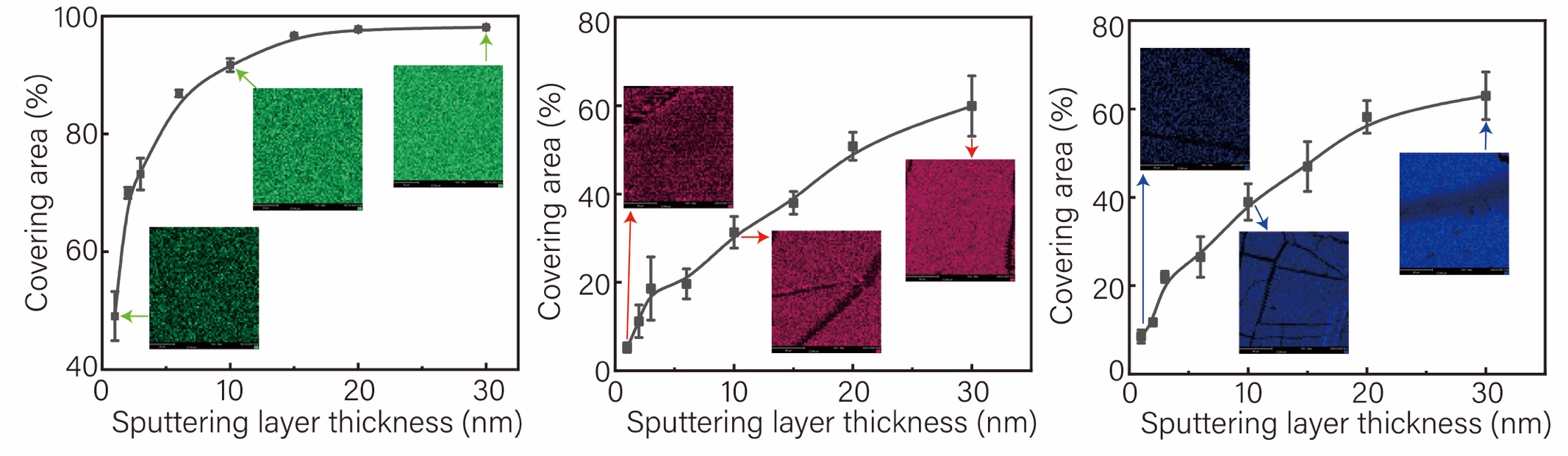


Figure S2. Au, Ag, and Pt layer covering area versus sputtering layer thickness.

## Static contact angle


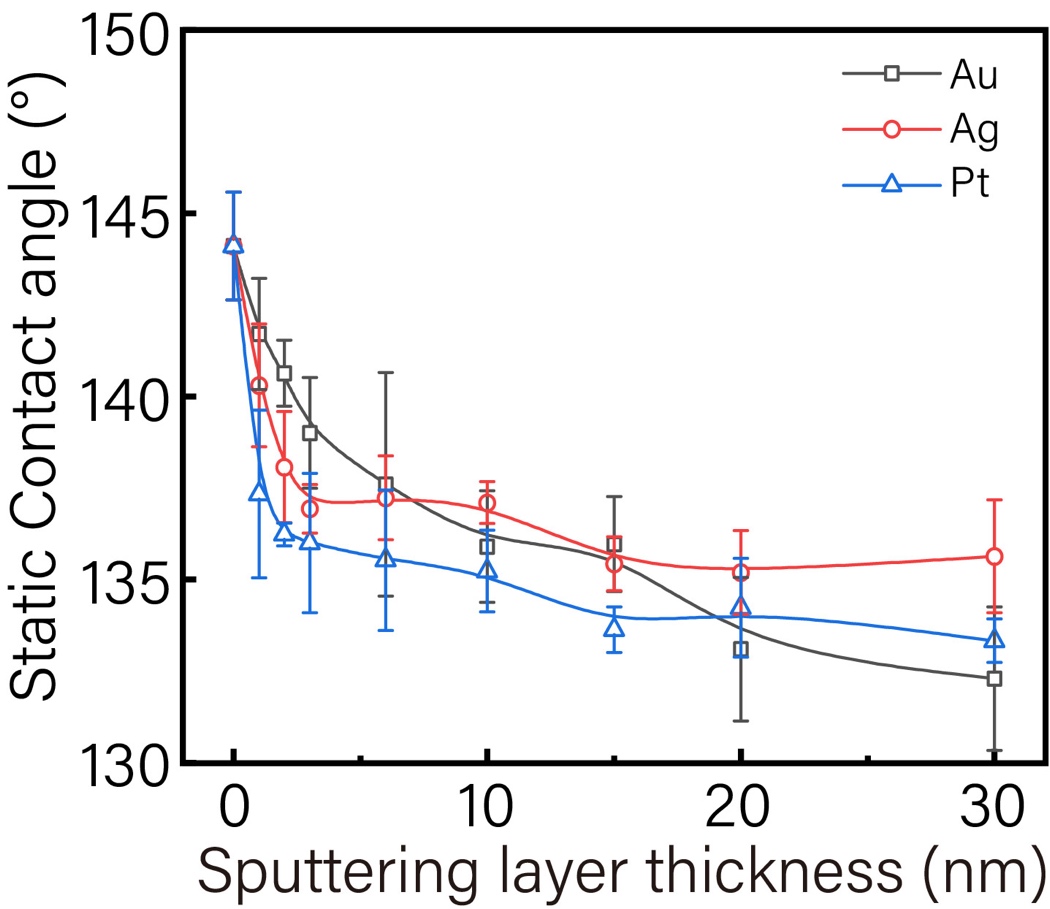


Figure S3. Static contact angle of EGaIn on Ecoflex surface. Metal sputtering is carried out with Au, Ag, and Pt separately. Metal sputtering times are 0, 10, 20, 30, 60, 100, 150, 200, and 300 seconds.

## Sliding angle


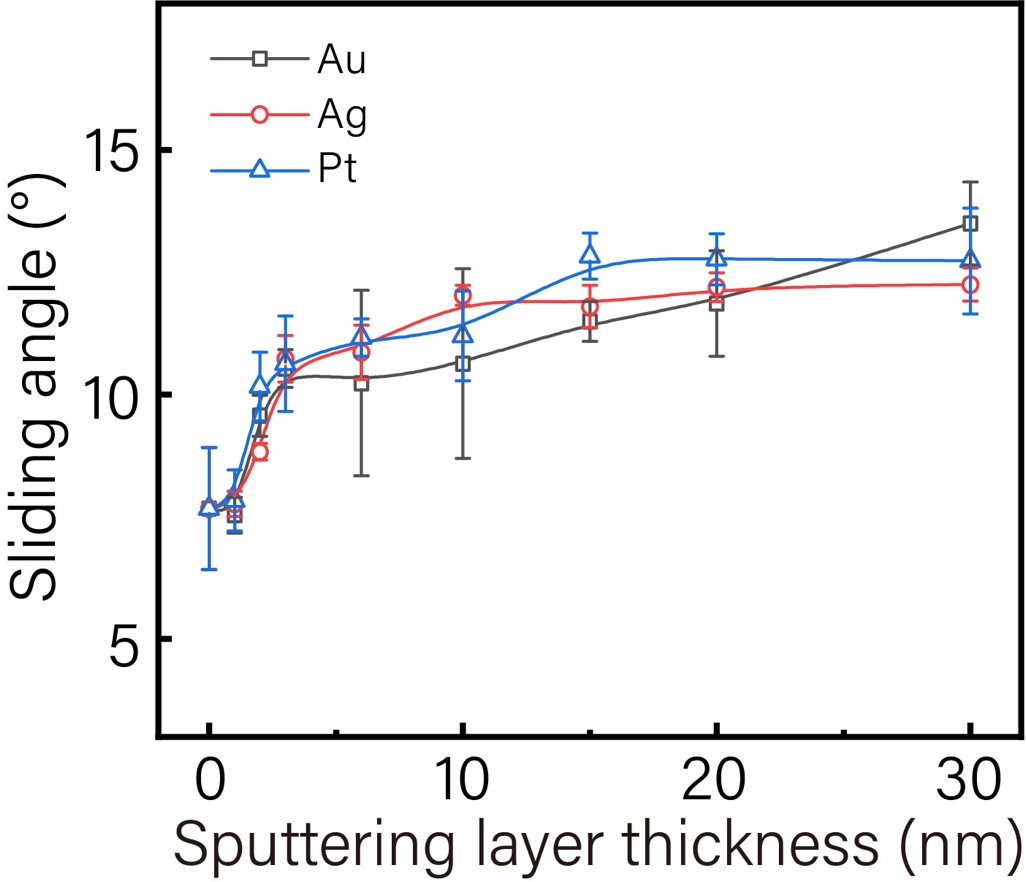


Figure S4. Sliding angle of EGaIn on Ecoflex surface. Metal sputtering is carried out with Au, Ag, and Pt separately. Metal sputtering times are 0, 10, 20, 30, 60, 100, 150, 200, and 300 seconds.

## Theoretical derivation of resistance variation


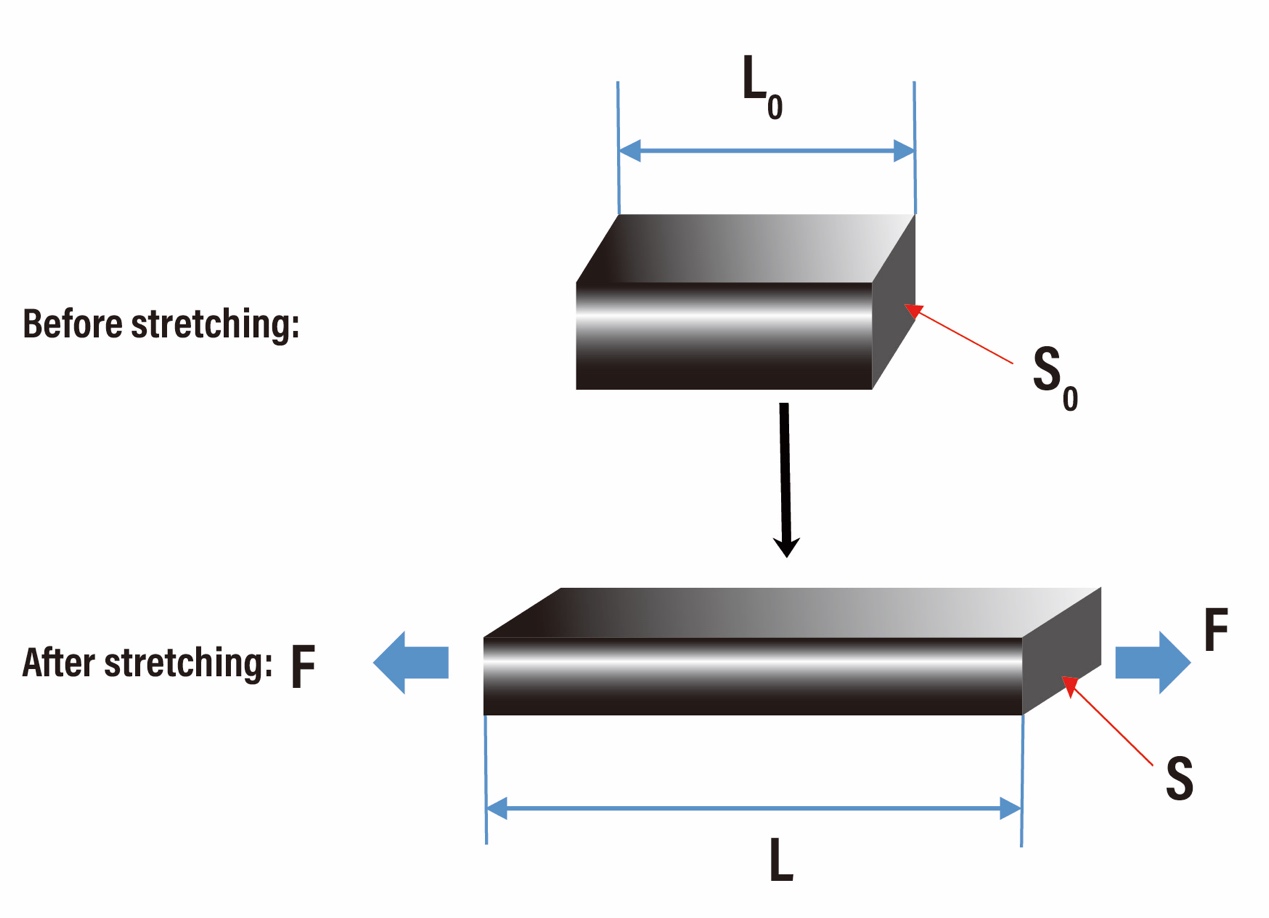


Figure S5. Theoretical model of resistance variation as a function of elongation ε.

Resistance law:

𝑅=𝜌 𝐿/𝑆

Volume formula:

𝑆=𝑉/𝐿

And,

$$\frac{R}{R_{0}}=\left( \frac{L}{L_{0}} \right)^{2}$$

To:

$$\boldsymbol{R=}\boldsymbol{R}_{\boldsymbol{0}}\left( \boldsymbol{1+\varepsilon} \right)^{\boldsymbol{2}}$$

## Loading–unloading experiment


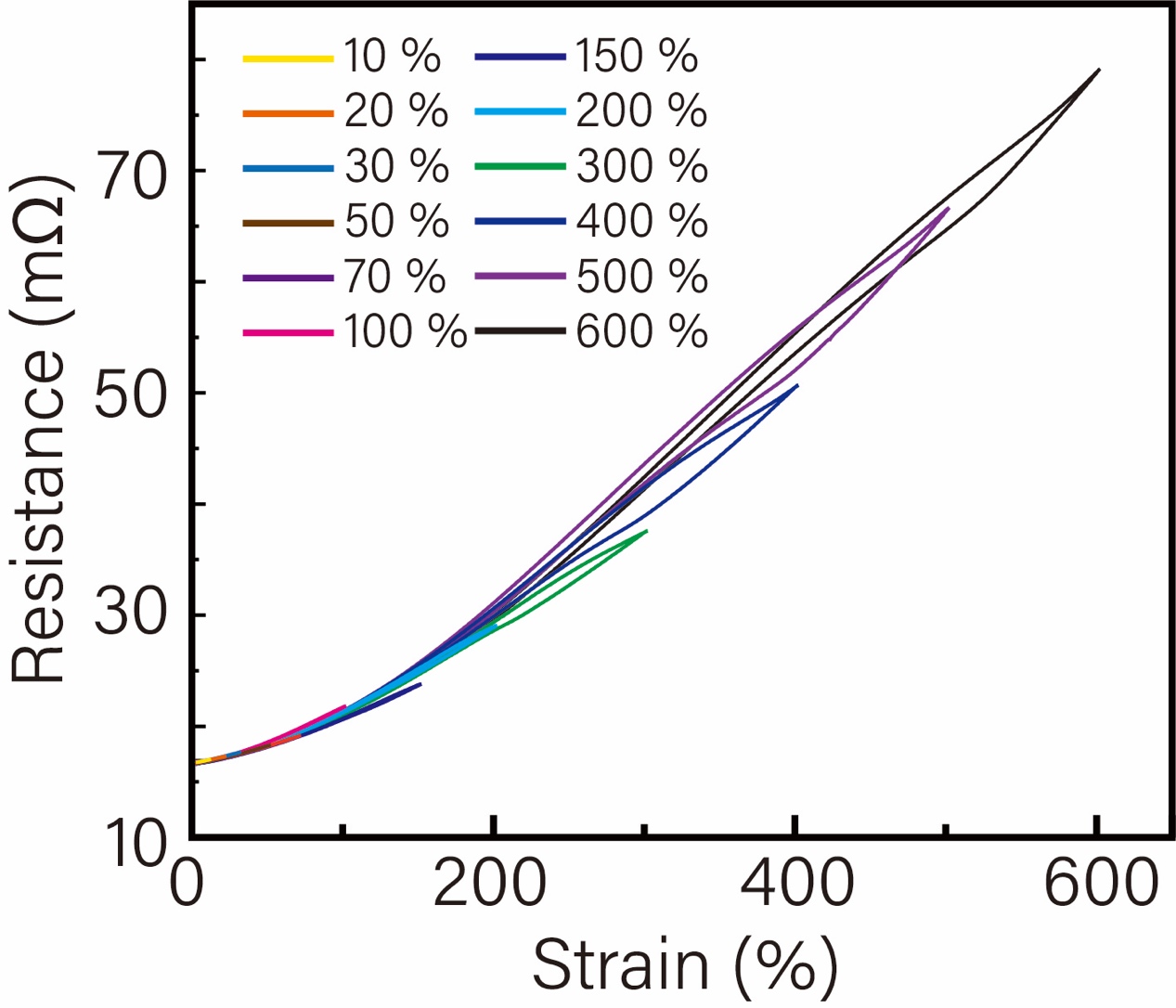


Figure S6. Loading–unloading experiments under different degrees of tension.

## Response to bending


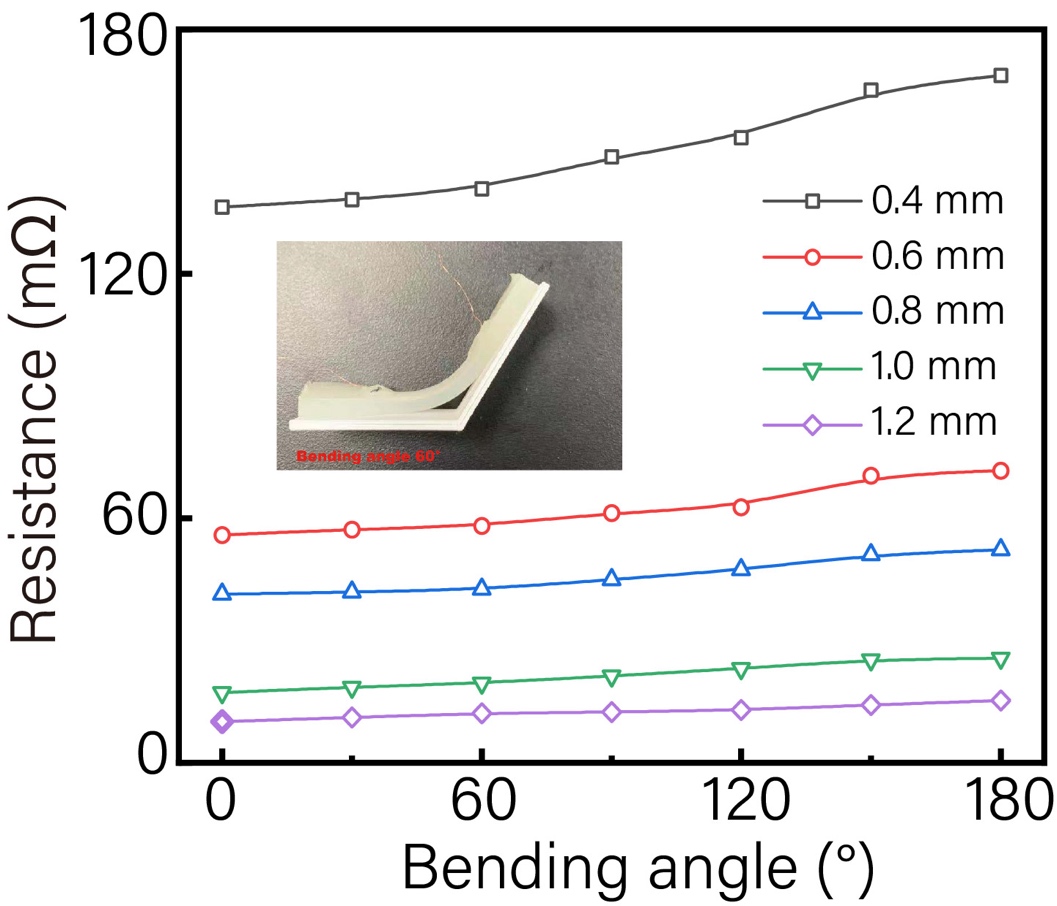


Figure S7. Bending tests and schematic diagram.

## Response to twisting


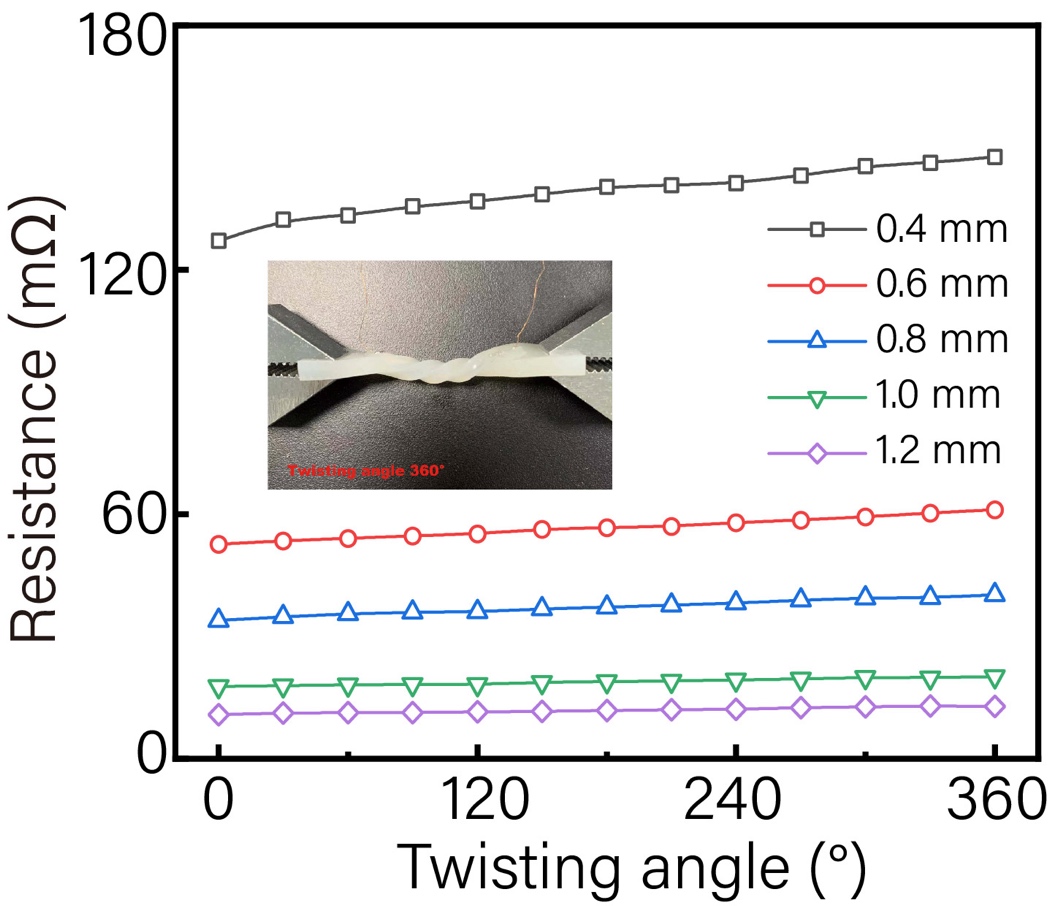


Figure S8. Twisting test and schematic diagram.

## Effect of channel size on circuit sensitivity


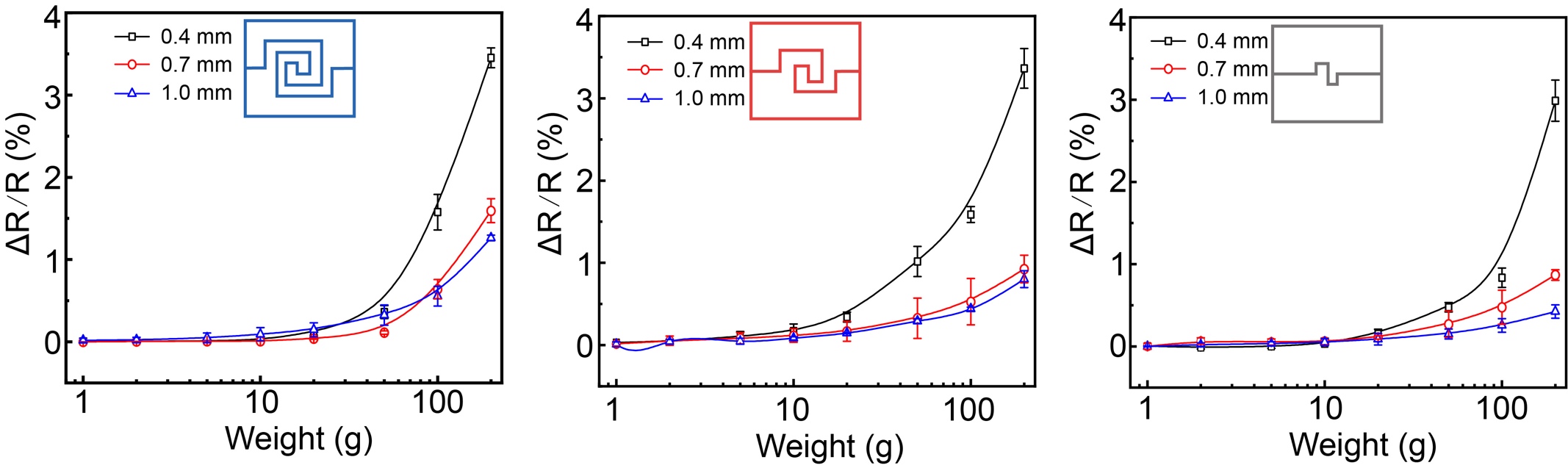


Figure S9. Effect of channel size on circuit sensitivity.

## Effect of substrate thickness on circuit sensitivity


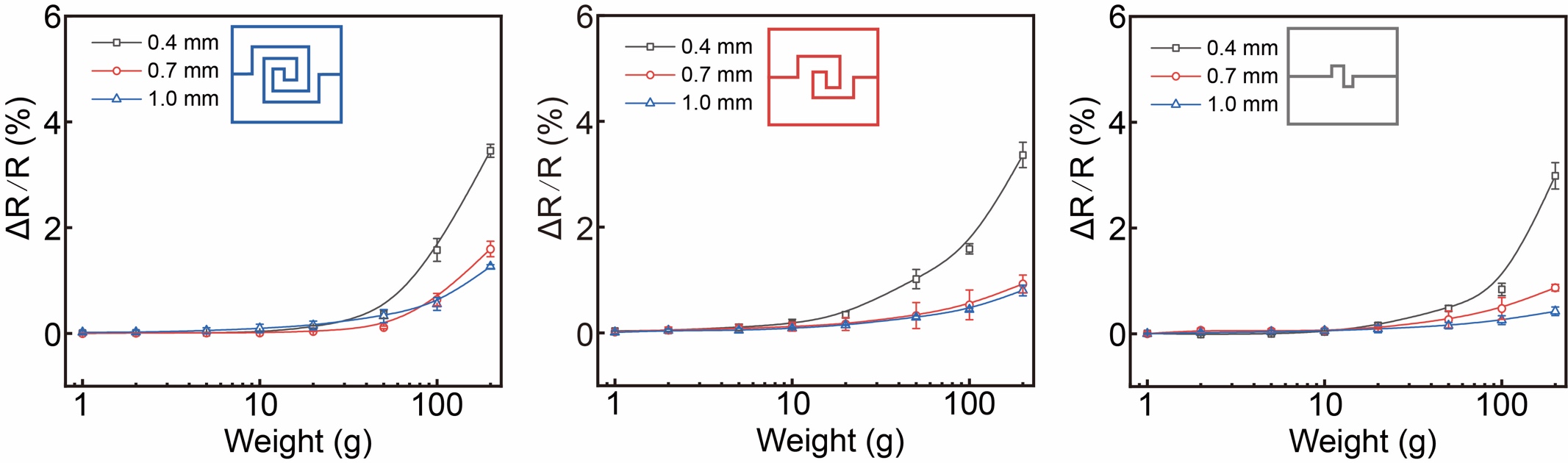


Figure S10. Effect of substrate thickness on circuit sensitivity.
